# Estimation of incidence and social cost of colon cancer due to nitrate in drinking water in the EU: a tentative cost-benefit assessment

**DOI:** 10.1186/1476-069X-9-58

**Published:** 2010-10-06

**Authors:** Hans JM van Grinsven, Ari Rabl, Theo M de Kok

**Affiliations:** 1 Dept. of Water, Agriculture and Food, Netherlands Environmental Assessment Agency, The Netherlands; 2 ARMINES/Ecole de Mines de Paris, France; 3 Dept. Health Risk Analysis and Toxicology, University of Maastricht, The Netherlands

## Abstract

**Background:**

Presently, health costs associated with nitrate in drinking water are uncertain and not quantified. This limits proper evaluation of current policies and measures for solving or preventing nitrate pollution of drinking water resources. The cost for society associated with nitrate is also relevant for integrated assessment of EU nitrogen policies taking a perspective of welfare optimization. The overarching question is at which nitrogen mitigation level the social cost of measures, including their consequence for availability of food and energy, matches the social benefit of these measures for human health and biodiversity.

**Methods:**

Epidemiological studies suggest colon cancer to be possibly associated with nitrate in drinking water. In this study risk increase for colon cancer is based on a case-control study for Iowa, which is extrapolated to assess the social cost for 11 EU member states by using data on cancer incidence, nitrogen leaching and drinking water supply in the EU. Health costs are provisionally compared with nitrate mitigation costs and social benefits of fertilizer use.

**Results:**

For above median meat consumption the risk of colon cancer doubles when exposed to drinking water exceeding 25 mg/L of nitrate (NO_3_) for more than ten years. We estimate the associated increase of incidence of colon cancer from nitrate contamination of groundwater based drinking water in EU11 at 3%. This corresponds to a population-averaged health loss of 2.9 euro per capita or 0.7 euro per kg of nitrate-N leaching from fertilizer.

**Conclusions:**

Our cost estimates indicate that current measures to prevent exceedance of 50 mg/L NO_3 _are probably beneficial for society and that a stricter nitrate limit and additional measures may be justified. The present assessment of social cost is uncertain because it considers only one type of cancer, it is based on one epidemiological study in Iowa, and involves various assumptions regarding exposure. Our results highlight the need for improved epidemiological studies.

## Background

### Integrated management of nitrogen cycle to improve welfare

Nitrogen is emitted to the environment by various sources in various forms that lead to a multitude of effects on human health, ecosystems and climate. On the other hand nitrogen is a key input for food production and deficient in many parts of the developing world. Therefore, Galloway et al. [1] conclude that "Optimizing the need for a key human resource while minimizing its negative consequences requires an integrated interdisciplinary approach and the development of strategies to decrease nitrogen-containing waste.". In fact the question is whether the N-cycle can be changed in such a way that a welfare improvement is achieved, implying that the economic benefits for society (the social benefits) from improvement of human health, ecosystems and climate should exceed the social costs of mitigation and their effect on prices of food or energy. Cost-Benefit Analysis (CBA) is one possible tool to assess welfare effects of environmental policies. However, CBA for integrated nitrogen policies is very complex in view of the many possible sources, effects and measures for nitrogen.

### Health impacts from nitrate in drinking water

The existence of adverse health impacts of nitrate via drinking water has been debated [2,3]. There are no assessments available of social costs of health impacts from emission of nitrate to drinking water. This is somewhat surprising as both in the EU and in the US policies and measures to remove or prevent nitrate in drinking water and groundwater have been in place for several decades mainly for reasons of preventive health care. Based on theoretical economic considerations, Hanley in 1990 [4] questioned the social benefits of blanket restrictions on nitrate use to implement the nitrate standard in the EC Drinking water Directive of 1980.

There is consensus that the role of nitrate exposure in causing methaemoglobinaemia is minor [5] and not a sound justification for the present nitrate standard for drinking water of 50 mg/L. However, significant adverse health effects from nitrate in drinking water are likely to result from complex interaction of exposure and cofactors affecting nitrite formation and nitrosation of amines and amides in the human body [5]. Adverse health effects related to nitrate or nitrite eventually, irrespective of its source, are caused by carcinogenesis from chronic exposure to N-nitroso-compounds. However, nitrate intake via drinking water is about four times [6] smaller than amounts from food or internal nitrogen metabolism. Nitrate intake also leads to increased physiological levels of nitric oxide which play a beneficial role in the vascular endothelial function and the defense against infections [7,8]. Nitric oxide and NO-synthase are also known to be involved in cancer-related events (angiogenesis, apoptosis, cell cycle, invasion, and metastasis) and are linked to increased oxidative stress and DNA damage [9].

Although there is evidence for both beneficial and adverse health effects of increased nitrate intake in drinking water, these effects are likely to be small and uncertain compared to other factors like life style and diet. Moreover, health effects may also results from other drinking water pollutants (pathogens, pesticides, trace metals). Therefore, it is not surprising that epidemiological studies into the relation between nitrate in drinking water and cancers (or other health effects) often provide weak associations, both positive and negative [5]. Ward et al. [5] conclude that: "The few epidemiologic studies that have evaluated intake of nitrosation precursors and/or nitrosation inhibitors have observed elevated risks for colon cancer and neural tube defects associated with drinking-water nitrate concentrations below the regulatory limit.". However, the European Food Safety Authority [6] concluded from a review of recent epidemiological studies, including the ones referred to by Ward et al., that: "However, these were mostly studies with a weak study design and limited strength of evidence; other case-control studies and cohort studies (which provide stronger evidence) find no increased risk with increasing nitrate intake after multivariate adjustment.". In response to L'hirondel et al. [10] who fundamentally reject the possibility of health risks from drinking water nitrate, Ward and De Kok [11] propose improved epidemiological study designs with longer time frames and evaluation of factors affecting nitrosation.

The present paper provides a method to assess the health costs by nitrates in drinking water. We compare the results with very rough estimates of the costs of improved water treatment and of reduced fertilizer use. However, the problem is more complex because of the multiple impacts and pathways of nitrogen in the environment. Nitrogen is a major factor for eutrophication and biodiversity loss and contributes to global warming. Even though most nitrates in drinking water come from fertilizer, a full analysis would have to consider more than just the passage from fertilizer to drinking water. Since part of the fertilizer nitrogen also ends up as emission of N_2_O, NO_2 _and NH_3 _to the atmosphere [12], the damage costs of the latter would have to be included in the search for optimal nitrate abatement - a point we take up again in the discussion.

## Methods

**Figure 1:**
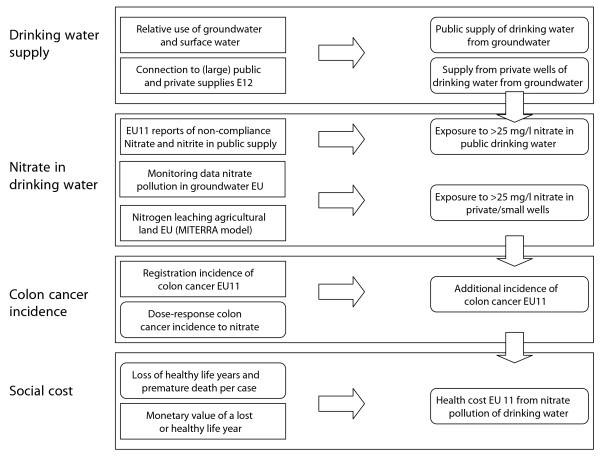
**Method to assess health damage by nitrate in drinking water in EU**. Schematic representation of a method to assess incidence and social cost of colon cancer induced by drinking water nitrate in the EU (rectangles for external data; rounded rectangles for assessment results).

For our assessment we assume a link with colon cancer as working hypothesis to provide a tentative assessment of health loss and social cost of nitrate in drinking water in the European Union. Ward et al. [5] indicate that colon cancer is the impact for which there seems to be more epidemiological evidence than for other types of cancer. In order to match the year of cancer registration data and monitoring data for nitrogen emissions and nitrate in groundwater and drinking water, we used data for the period 1995-2000. The procedure included the following steps (see also Figure 1).

### Present incidence and prevalence of colon cancer in Europe

Data were taken from IARC [13], Micheli et al. [14] and Boyle and Ferlay [15]. Cancer registration data refer to the mid-1990's. Incidence (World standard age-adjusted) of colon cancer in 1992 in Europe [13] was 17 cases per year (per 100,000), prevalence was 176 cases (per 100,000), making colon cancer one of the most frequent cancer sites. Boyle and Ferlay [15] extrapolated these data to 2004 and estimated incident cases of cases colon+rectum cancer in EU25 at 279,000 and mortality at 139,000, inferring a mortality rate of nearly 50%. Risk factors for colon cancer are only partly understood. Diet and physical activity are probable risk factors for colon cancer, while a minor fraction of risk is hereditary. In many countries in the EU, colon cancer incidence increases with time because populations are ageing and incidence increases with age. This effect is not fully compensated by improved diagnosis and medical treatment. Incidence of colon cancer varies considerably over Europe with low values in Finland, Poland and Baltic states (10-13 new cases per 100,000 per year) and high values in Germany, Czech Republic, UK and Italy (23-24 new cases per 100,000 per year) [13].

### Loss of healthy life years and life years from premature death for colon cancer

We inferred loss of healthy life years and life years due to premature death using data on colon cancer incidence and mortality per five year age class between 1989 and 2006 provided by the Dutch Cancer Registry (http://www.ikcnet.nl/). Total prevalence of colon cancer in the Netherlands, 169 cases per 100,000, compares well to the average situation in the Europe, 176 cases per 100,000 [14]. Incidence difference between sexes is small and therefore was not considered. The average age (weighted by incidence per age class) of a colon cancer patient between 1995 and 2000 was determined at 70.4 years, and age of death at 74.0 years. Average life expectancy between 1995 and 2000 was 77.8 years, implying an average loss of life for fatal colon cancers (mortality rate is 43% five years after diagnosis) in the Netherlands of 3.8 YLL (Years of Life Lost). Years Lived with Disability (YLD), i.e. loss of healthy life years for all patients, including those that are cured, was calculated as the ratio of prevalence over incidence. Using prevalence data of IARC [13], we found a YLD of 4.9 for the Netherlands, and we assume that value for the other countries as well.

### Increased risk of colon cancer due to exceedance of the nitrate standard in drinking water

We derive the increased colon cancer risk from DeRoos et al. [16], a case-control study on nitrate in public water supplies in Iowa. This study did not find an association between colon cancer and nitrate for the total population, but did find such associations for specific subgroups. In particular the subgroup with above median meat intake showed an association with nitrate. For the subgroup with more than 10 years of exposure to nitrate concentrations exceeding 5 mg/L of NO_3_-N (22 mg/L NO_3_, which is half the legal US limit and approximately half the WHO and EU nitrate standard of 50 mg/L), the odds ratio almost doubled as compared to the reference group that was not exposed to NO_3_-N levels exceeding 5 mg/L. This association was not found for rectum cancer.

**Figure 2:**
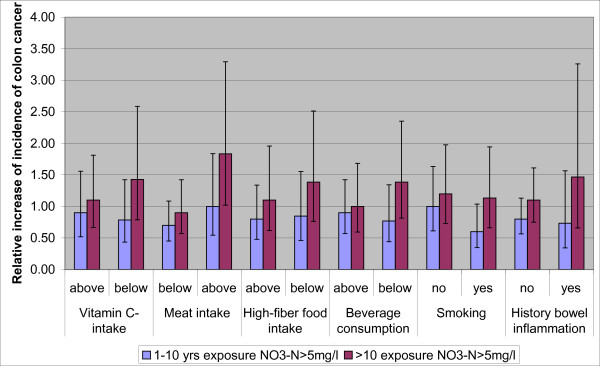
**Increase of colon cancer incidence by drinking water nitrate in Iowa**. Increase of incidence of colon cancer (and 95% confidence intervals) in Iowa public water supply, for subgroups with above and below median dietary and medical risk factors, and with 1-10 years of exposure and more than 10 years of exposure to NO_3_-concentrations in drinking water exceeding 25 mg/L NO_3_, relative to the subgroup with no exposure (after [14]).

Figure 2 shows for almost all dietary and medical groups an increased risk of colon cancer when exposed to NO_3_-N exceeding 5 mg/L for more than ten years, but only the increased risk of approximately a factor 2 for the subpopulation with above median meat intake is statistically significant (95% CI). For the purpose of this assessment we assumed a doubling of colon cancer incidence for above median meat consumers exposed to a NO_3_-N concentration exceeding 5 mg/L (22.5 mg/L NO_3_). In an ecologic study, Gulis et al. [17] found an increased incidence of colorectal cancers by 66% in the total population in the Trnava District in Slovakia, above NO_3_-N concentrations of 4.5 mg/L in drinking water as compared to a reference group below 2.2 mg/L. These results are similar to those of DeRoos [16], for which a 50% increase of colon cancer incidence can be inferred for the total population, and considering that Gulis et al. [17] combined colon and rectum cancers. In an ecologic study in the province of Valencia in Spain, Morales-Suarez-Varela et al. [18] did not find a positive association with mortality from colorectal cancers for NO_3_-N concentrations exceeding 2.2 mg/L, but did find nearly a doubling of the relative risk of mortality for stomach cancer. Gulis et al. [17] state that these different results may be caused by using mortality data, which are a function of both incidence and changes in survival (e.g. because of developments in medical diagnosis and treatment). The studies by Gulis et al. and Morales-Suarez-Varela et al. did not consider dietary or medical risk factors.

### Relationship between nitrogen leaching and nitrate in groundwater

First, we investigated potential associations between observed nitrate concentrations in shallow aquifers and estimates of the nitrogen leaching from agricultural land. For this purpose we combined monitoring data reported by Zwart et al. [19] to the EU-commission^2^, with model estimates of the agricultural nitrogen leaching from the rooting zone by Velthof et al. [12]. Velthof et al. use the MITERRA-EUROPE model which calculates N-leaching as a fraction of the N-surplus. This leaching fraction depends on soil type, land use, organic matter content, precipitation surplus, temperature and rooting depth. It should be pointed out that monitoring procedures in different EU member states may vary considerably, e.g. with respect to depth and frequency and density of sampling [20]. The median depth for monitoring of implementation of the EU Nitrates Directive is between 10 and 20 m (based on 22,000 samples for 18 EU member states taken between 2004 and 2007; data come from Member State reporting to the European Commission as referred to in art. 10 of the Nitrates Directive (91/676/EEC). We used the data for sampling depths between 5 and 20 m, which are common extraction depths for private wells (judging from procedures on websites of private constructors).

**Figure 3:**
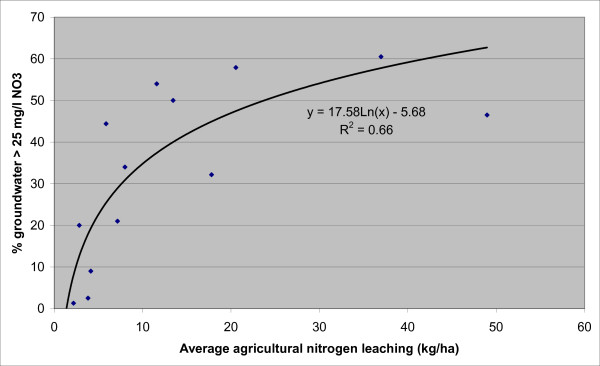
**Groundwater in EU exceeding 25 mg/L nitrate**. Data points and logarithmic fit for fraction of groundwater samples that exceed 25 mg/L NO_3_, as function of modelled leaching of nitrogen from agricultural soils.

A logarithmic relation (R^2 ^= 0.67) was found between the % of samples with exceedance and the mean nitrogen leaching intensity (kg/ha/yr) (Figure 3; eq. 1).

(1) LAE=17.57⋅ln(N−leach)–5.67

where LAE = fraction of land area in country with exceedance and N-leach = mean nitrogen leaching intensity (kg/ha/yr). We assume the percentage of exceedance in monitoring to be proportional to the land area where 25 mg/L NO_3 _was exceeded and therefore also a proxy for the percentage of the population, using private wells or small communal supplies, that is exposed to drinking water exceeding 25 mg/L NO_3_. We did not consider temporal trends of nitrate in groundwater.

### Relationship between exceedance of critical nitrate concentration in groundwater and public exposure to drinking water exceeding the critical limit

The relationship between nitrate in groundwater and in drinking water depends on the drinking water infrastructure and water treatment in the different EU member states. Important variables are the percentage of the population connected to public supply, the presence of drinking water treatment in public supplies and the relative use of surface water and groundwater. These parameters vary considerably across the EU; for 12 member states, selected for data availability, connection to large public supplies (serving more than 5000 customers or delivering more than on million liters per day) ranged from 36 to 100% and use of groundwater for public supply from 25 to 99% (Table 1; [21]). Exposure to drinking water exceeding 25 mg/L NO_3 _may result from incidental exceedance in public supplies or structural exceedance in small local facilities or private wells.

**Table 1:** Drinking water supply and exposure to >25 mg/L nitrate

	Population	Connected to large public water supply	Groundwater based water supply	Non-compliance of large public supplies with EU nitrate or nitrite standard	Population exposed to groundwater based large public supply with >25 mg/L NO_3_	Agricultural land	N-leaching to groundwater	Area with groundwater exceeding 25 mg/L NO_3_	Population exposed to small or private groundwater wells with >25 mg/L NO_3_
	million	%	%	%	%	%	kg/ha/yr	%	%
Austria	7.8	60	95	2.0	2.0	41	4	18	6.8
Belgium	10.2	90	53	1.0	2.8	46	37	58	3.1
Denmark	5.3	74	99	0.3	3.3	62	24	50	12.9
Finland	5.3	36	34	4.5	0.7	7	3	13	2.7
France	59.7	73	64	2.4	2.7	54	13	40	7.0
Germany	82.7	82	72	1.4	3.1	49	18	45	5.8
Greece	10.7	69	50	0.0	0.6	66	4	19	3.0
Ireland	3.6	75	25	0.0	0.6	23	11	36	2.3
Italy	57.6	83	85	0.7	2.3	53	8	31	4.5
Netherlands	15.9	100	66	0.0	3.5	58	49	63	0.0
Spain	39.6	73	35	3.5	1.4	60	6	25	2.4
UK	58.1	98	27	3.7	1.8	70	12	37	0.2
EU12	356	60	81	2.0	2.3	50	11	34	4.1

Drinking water supply, nitrogen loading, nitrate leaching and exposure to drinking water exceeding 25 mg/L NO_3 _in the period 1995-2000 in 12 EU member states selected for availability of data on drinking water infrastructure and quality.

Non-compliance with EU legal limits in the EU drinking water directive [22] for either nitrate (50 mg/L NO_3_) or nitrite (0.5 mg/L NO_2_) between 1995 and 2000 occurred in 0 and 4.5% of the water samples for large supplies in 12 reporting EU member states [21]. Non-compliance between 2000 and 2004 in 14 reporting member states was somewhat lower, but in the same range, with a highest level of non-compliance to nitrite of 11% in Denmark [23].

It may be expected that exposure in eastern European countries is higher than in northern and western Europe in view of a lower access of the rural population to improved drinking water supply (10-70% [24]) and lower implementation levels of environmental policies.

In the Netherlands, nitrate concentrations in drinking water exceeding 25 mg/L NO_3 _in 2001 were reported [25] in 5% of a total of 218 public drinking water production sites using groundwater, while there was no violation of the legal limit of 50 mg/L NO_3_. Data on exceedance of 25 mg/L NO_3 _for the other 11 member states were not available and estimated using the Dutch value and the ratio of exceedance of 25 mg/L NO_3 _in untreated groundwater. Next exposure to drinking water from groundwater in public and private supplies exceeding 25 mg/L NO_3 _and using groundwater can be calculated (eqs. 2 and 3).

(2) $${\text{Pop}}_{\text{L}}=\;\left(\text{Exc EU}+\text{Exc 25 mg}/\text{L}\right)\cdot \text{Grw}\cdot \text{Connect}$$

where Pop_L _= fraction (%) of population in country exposed through large public supply systems; Exc EU = sum of levels (%) of non compliance to 50 mg NO_3 _and 0.1 mg/L NO_2 _for drinking water samples between 1995 and 2000 as officially reported to EU; Exc 25 mg/L = estimated fraction (%) of drinking water with NO_3 _between 25 and 50 mg/L taking the exceedance value of 5.3% for the Netherlands (NL) and assuming that Exc 25 mg/L is proportional to LAE_country_/LAE_NL_; Connect = fraction (%) of population connected to large public supply; and Grw = fraction (%) of drinking water production from groundwater.

(3) $${\text{Pop}}_{\text{S}}=\text{Grw}\cdot \left(\text{1}-\text{Connect}\right)\cdot \text{LAE}$$

where Pop_S _= fraction (%) of population in country exposed through private wells and small public supply systems.

### Nitrate associated additional colon cancer cases in the EU

First the total colon cancer incidence was taken from section 'Present incidence and prevalence of colon cancer in Europe'. Next the proportion of cases associated with nitrate was inferred from section 'Increased risk of colon cancer due to exceedance of the nitrate standard in drinking water', where we assume that of the population exposed to NO_3 _concentrations above 25 mg/L the half that consumes more than the median amount of meat, has a risk of colon that is twice as high as for the total population (eq. 4).

(4) $$\Delta \text{Incidence}=\Delta \text{R}\cdot \text{Incidence}\cdot \left({\text{Pop}}_{\text{S}}+{\text{Pop}}_{\text{L}}\right)/\text{2}$$

where Δ Incidence = additional incidence of colon cancer due to exceedance of 25 mg/L NO_3 _in groundwater based drinking water; Incidence = crude total incidence of colon cancer (using 1993-1997 crude rates by IARC per 100,000); ΔR = increased risk of colon cancer for individuals that have consumed drinking water with NO_3 _in excess of 25 mg/L longer than 10 years and with above median meat consumption (ΔR = 2-1 = 1; see Figure 2); division by 2 to account that only half of the exposed population is an above median meat consumer.

Implicitly we assume that the association with meat consumption in the EU is the same as in Iowa and that the duration of exposure above 25 mg/L NO_3 _was more than 10 years.

### Economic valuation of cancers

We base the valuation of cancers on the number of years of life lost (YLL) and the number of years lived with disability (YLD), i.e. the loss of healthy life years. Thus we combine the nitrate related additional colon cases with the result of the section 'Loss of healthy life years and life years from premature death for colon cancer' to calculate the YLD and YLL both for individual member states and the EU (eq. 5).

(5) Soc-Cost=ΔIncidence⋅(YLD⋅VYLD+YLL⋅VOLY)

where Soc-Cost = social cost of loss of healthy life years and premature death from additional colon cancer; YLD = Years of life lived with disease (per colon cancer case); VYLD = value of a YLD = QALY score - VOLY; YLL = years of life lost (per colon cancer case); and VOLY = economic value of a life year.

For the value of a life year (VOLY) we take 40,000 euro/YLL, as determined by a large contingent valuation study in nine EU countries by Desaigues et al. [26]; that value is now used by the ExternE project series, the European program to assess the external costs of pollution [27].

For the valuation of years lived with disability (YLD) we invoke the DALY and QALY scores that have been published for colon cancer. The DALY (Disability Adjusted Life Year) is an indicator for the severity of a health condition. Developed by the World Health Organization, the DALY is a number between 0 (perfect health) and 1 (death). The QALY (Quality Adjusted Life Year) is a similar indicator, but its range is opposite, from 0 (death) to 1 (perfect health). A DALY is roughly equivalent to 1 - QALY, although their precise definitions involve differences such as discounting and age-weighting (for DALY but not QALY). Such differences do not matter in view of the uncertainties, and we set the monetary value of a DALY or QALY equal to 1 VOLY = 40,000 euro/YLL. Mathers et al. [28] indicate a DALY score of 0.2 for colon cancer during the period of diagnosis/treatment/waiting. For the QALY scores of colon cancer CEA [29] cites numbers ranging from 0.5 to 0.74 with a mean of 0.64. In view of these scores we take 0.3 as an approximate mean of 0.2 and 0.36 = 1 - 0.64, and thus we value each year lived with colon cancer as 0.3 VOLY = 12,000 euro/YLD.

Needless to say, the uncertainties of the monetary valuation are large. In particular we note that often an alternative approach is used for the monetary valuation of fatal cancers, based on the value of a prevented fatality (also known under the unfortunate name "value of statistical life") for which the DG Environment of the European Commission assumes approximately one million euro [30]; some economists even add a cancer premium of about 50 to 100% to that because of the dreaded nature of this disease. The result is a cost in the range of 1 to 2 million euro per fatal cancer. There is no consensus on which approach is best, and the valuation of fatal cancers could be an order of magnitude higher than what we obtain by using YLL. In view of such uncertainties the cost of medical treatment is insignificant even though in a more definitive assessment it should also be included.

### Calculation of unit N-costs

A unit N-cost or -benefit is defined as the monetary value of an effect (adverse or beneficial) expressed per kg of pollutant or kg N in pollutant or per kg N in applied fertilizer. The unit N-cost approach allows a first comparison of the social benefit of less nitrate in drinking water to the social cost of measures to mitigate nitrate e.g. by water treatment or reduction of fertilizer use (eq. 6).

(6) UCN=Soc-Cost/N-loss

where UCN = Unit damage cost (euro per kg of N leaching); and N-loss = nitrogen leaching loss from agricultural land (kg).

## Results

### Exposure to nitrate in drinking water

Connection to large public water supply varies considerably and is, among other factors, related to population density, cost for installing drinking water infrastructure and national policies. Also the use of groundwater for drinking water varies and is typically related to the presence of aquifers. Using Equation 1 (Figure 3), the area with groundwater exceeding 25 mg/L NO_3 _ranged between 20% and 60% (Table 1). About two-thirds of exposure to drinking water with concentrations exceeding 25 mg/L NO_3 _comes from private supply and small facilities. Estimates of the exposed population using small or private wells ranges from 0% in countries with nearly 100% supply through large facilities (Netherlands and UK), to nearly 13% in Denmark. For the 12 EU member states, the total exposed population amounts to 23 million persons (6.5% of total population) of which 8 million persons (2.3%) were exposed through public supply.

### Morbidity, mortality and health loss due to nitrate induced colon cancer

In Table 2 we estimate the number of additional colon cancer cases for the 11 member states (excluding Greece because no cancer registration data were available) at 4800 per year (3.4% of total incidence) using the assumption based on DeRoos et al. [16] that for individuals with above median meat consumption, exposure to drinking water with > 25 mg/L NO_3 _for more than 10 years doubles the risk. The total loss for these 11 countries is 23,000 YLD and 18,000 YLL. Although this loss is modest, it represents a total social cost of 1.0 billion euro per year or 2.9 euro year per person averaged over the entire population (corresponding to three days of additional morbidity and mortality in an average life), and to 150 euro per year for a person exposed to drinking water exceeding 25 mg/L NO_3_. Low values (less than one euro/capita) are found for the UK, Finland and Ireland and in part could be viewed as benefits of investments in a good drinking water infrastructure. The highest values (3-7 euro/capita) are found for Denmark, Italy, France and Germany, in part due to lower levels of connection to or availability of large high quality drinking water infrastructure, in combination with high nitrate leaching. Finally the unit cost is obtained by dividing the health cost by the total quantity of NO_3_-N leaching in each country. Unit damage cost for the 11 countries ranges between 0.1 and 2.4 euro per kg of N leaching, with an average of 0.7 euro/kg.

**Table 2:** Health damage from drinking water nitrate related colon cancer

	Total population exposed to >25 mg/L NO_3_	Total incidence of colon cancer (1993-1997)	Additional colon cancer cases due to nitrate per year	Total number of lost healthy life years	Total number of lost life years from premature death	Monetary value of loss of (healthy) life years		Unit health damage cost from N-leaching agricultural land
	%	x1000	x1000	x1000	x1000	million euro/year	euro/capita	euro/kg
Austria	8.8	2.5	0.1	0.5	0.4	23	2.9	1.9
Belgium	3.1	3.7	0.1	0.5	0.4	23	2.2	2.4
Denmark	16.2	2.0	0.2	0.8	0.6	35	6.6	0.6
Finland	3.4	1.2	0.0	0.1	0.1	4	0.9	0.8
France	9.7	19.8	1.0	4.7	3.6	202	3.4	0.6
Germany	8.9	42.0	1.9	9.1	7.1	393	4.8	1.4
Ireland	2.8	1.1	0.0	0.1	0.1	3	0.9	0.1
Italy	6.8	28.3	1.0	4.7	3.6	202	3.5	1.9
Netherlands	3.5	5.5	0.1	0.5	0.4	20	1.3	0.2
Spain	3.8	12.6	0.2	1.2	0.9	51	1.3	0.4
UK	2.0	20.4	0.2	1.0	0.8	43	0.7	0.2
EU11	6.5	139	5	23	18	1000	2.9	0.7

Increased incidence of colon cancer due to nitrate in drinking water from groundwater, the associated loss of healthy life years and loss of life due to premature death and the monetary valuation of this loss for 11 EU member states (population 345 million).

## Discussion

### Uncertainty

The results for social cost and unit cost of health loss due to nitrate in drinking water should be viewed as tentative values for comparative use against social costs or benefits of impacts for other nitrogen pollutants or against cost of measures. In fact, our assessment is based on just one epidemiological study in Iowa using a number of educated assumptions and guesses about exposure in the EU. The main sources of data uncertainty are discussed in Table 3.

**Table 3:** Overview and discussion of major sources of data uncertainty

**Source of uncertainty**	**Evaluation**
Exposure-response function	Epidemiological evidence is suggestive but far from conclusive.
Differences in water supply and life style factors for colon cancer incidence between Iowa and Europe.	Meat intake is an important risk factor for cancers. Total meat consumption in Europe and the US are comparable, but beef consumption in Iowa is higher.
The assumption that exceedance of 25 mg/L NO_3 _in groundwater samples at 5-20 m depth is equivalent to exposure in all drinking water from small public supply and private wells.	No data were available about extraction depth and water treatment for this type of supply. Local data on nitrate in groundwater and actual use for drinking water were not available.
Focus on groundwater that is affected by agricultural nitrogen loading.	Relatively unpolluted aquifers overlain by forest or semi-natural vegetation are underrepresented. Therefore exposure probably is overestimated.
Not considering surface water based drinking.	Considering non-compliance in surface water based public drinking water increases health cost by about 15%. Although about 40% of EU surface waters exceed 25 mg/L NO_3 _[41], we assume that private use of surface water for drinking water is negligible compared to groundwater.
Not considering consumption of bottled water.	In EU27, the consumption of bottled drinking water, that is very low in nitrate, increased from around 12% of total intake in 2001 to 15% in 2007 and consideration would slightly lower exposure estimates. In fact total beverage consumption is relevant; fruit juices can be high in nitrate and beers high in nitrosamines [42].
The assumption that percentage of drinking water samples from large public facilities not complying with standards for nitrate (50 mg/L) or nitrite (0.5 mg/L NO_2_) is equivalent to exposure, and identical for groundwater and surface water sources	Non-compliance may be incidental and assumption may overestimate exposure. Estimates of exposure to exceedance of 25 mg/L NO_3 _will be more robust.

In view of the uncertainty about the health impact itself, the lower limit of the health cost is zero, and in line with the lower limit of the 95% confidence interval of the risk increase inferred from DeRoos et al. [16]. Using the subgroup with above median meat consumption to quantify the risk increase of colon cancer, could be regarded as a worst case approach. On the other hand colon cancer is just one of over ten possible cancers for which positive, but also negative, associations with nitrate have been published [5]. A further illustration of the complexity of the relation between nitrogen in drinking water and human health is the production of the potent carcinogen N-nitrosodimethylamine (NDMA) caused by interaction of disinfection treatment of drinking water sources and environmental concentrations and mixtures of unknown nitrosamine precursors [31].

Another source of bias is that our analysis is based on data for 11 "old" EU member states with relatively high GDP and levels of implementation of environmental and drinking water policies. For the new central and east EU member states the social cost per capita is expected to be higher.

### Potential health benefits and mitigation costs

Typical measures to prevent nitrate exceedance in drinking water are blending polluted water with clean water, biochemical water treatment and installing deeper extraction wells. Data on costs of these measures are scarce but the costs are expected to decrease with increasing scale of the drinking water production. Illustrative annual cost values are 0.5 euro/capita/yr for water treatment and mixing for the UK and the Netherlands where large aquifers are available [32,33], 3 euro/capita/yr for Austria and Germany when also extraction wells or drinking water infrastructure need adjustment [34,35] and cost can be as high as 15 euro/capita/yr when new private wells are required (based on internet information from contractors in the USA). Potential health benefits for the UK and the Netherlands (0.7 and 1.3 euro/capita/yr respectively) appear to be higher than present treatment costs. For Austria and Germany (3 and 5 euro/capita/yr respectively) there are also potential welfare gains even for installation of shallow, less expensive types of private wells. Hanley [4] concluded for East Anglia in the UK that in 1989 the willingness of households to pay (WTP) for drinking water not exceeding the EU nitrate standard was larger than the actual costs of the required treatment. Our results support Hanley's conclusion, however, it is doubtful whether these potential gains would motivate public authorities or individuals to invest in nitrate treatment or drinking water infrastructure, as the costs may appear more tangible than the potential health gains.

### Potential health benefits and nitrogen fertilizer

Areas with aquifers suitable for groundwater extraction for drinking water production often are also areas suitable for agriculture. For this reason use of fertiliser or manures is a major source of nitrate pollution, and reduction of this use is a typical measure to prevent nitrate pollution of aquifers. Agricultural production clearly benefits from additional nitrogen input, but there is an optimum and for some crops (e.g. cereals) the yield diminishes when the nitrogen input is further increased (see for example Lord and Mitchell [36]). Although unit cost analysis for agricultural production is complex and beyond the scope of this paper, we give one example of a possible outcome for winter wheat, which is the most important food crop in the EU (Table 4). Around typical nitrogen fertilizer input levels for north-western Europe between 100 and 200 kg/ha/yr the marginal economic return for a farmer on fertilizer-N ranges between 1 and 3 euro/kg of N (fertilizer prices between 2000-2006 were 0.6-0.8 euro/kg of N; wheat prices 125 euro/ton and yield response to N ranging between 10-35 kg wheat/per kg). For sandy-loamy soils, about 10-20% of this input leaches to the typical extraction depth for private wells of 5-20 m. Results in Table 4 show that on average health costs associated with nitrate leaching (0.15 euro/kg of N) reduce net benefits of fertilizer use (1.8 euro/kg of N) by less than 10%. However, at the lower end of the range health costs (0.5 euro/kg of N) are comparable to the benefits (0.6 euro/kg of N). Values of N-benefits of 0.6 euro/kg, or lower, represent regions with a weak yield response to N-fertilizer (e.g. the northwest of Europe where crop yields are close to their maximum, and the south and east of Europe where yields are often limited by water shortage), while high values of N health costs represent regions where drinking water production is vulnerable to nitrate leaching (Austria, Belgium, Germany, Italy).

**Table 4:** Tentative comparison of costs and benefits of fertilizer use

	Emission	Unit cost value	Unit benefit value	Net unit benefit
	kg/ha/yr N	euro/kg N-use or N-emission		euro/kg N-fertilizer
Nitrogen fertilizer use^1^	100 - 200	0.6 - 0.8 (0.7)	1.2 - 3.5 (2.5) ^3^	0.6 - 2.7 (1.8)
		Health costs^2^		
Nitrate leaching	10 - 40	0.1 - 2.4 (0.7)		-0.5 - 0.0 (-0.15)
Nitrogen oxide emission to air	0.2 - 1.2	2 - 32 (20)		-0.2 - 0.0 (-0.12)
Ammonium emission to air	1 - 6	2 - 36 (12)		-1.1 - 0.0 (-0.35)
Total				0.6 - 1.0 (1.3)

Comparison of the health cost of nitrate leaching with the social benefit of fertilizer use, and with health costs of emissions of nitrogen oxides and ammonia from fertilizer (ranges and, in between brackets, mean values).

^1)^Use of CAN (calcium ammonium nitrate) on winter wheat.

^2) ^Range refers to range of values for EU member states.

^3)^Inferred from nine data sets of field trials across Europe.

Since part of the fertilizer or manure nitrogen ends up as emission of N_2_O, NO_2 _and NH_3 _to the atmosphere [12], the damage costs of the latter should also be included in a CBA for N-fertilization. These damage costs have been assessed recently for Europe in two research programs, ExternE [26] and CAFE [37], in the USA by US EPA [38], and by the World Health Organization [39]. These assessments are fairly consistent with each other since the underlying assumptions are very similar. Recent estimates by ExternE of health damage costs are 5.6 euro/kg of NO_x _(equivalent to 20 euro/kg of N) and 9.5 euro/kg of ammonia (equivalent to 11.6 euro/kg of N). In ExternE the exposure-response functions for health are assumed to be linear without threshold. Health impacts of these compounds are mainly indirect and mediated through several steps, where NO_x_, and NH_3 _act as precursors for ozone and/or airborne particulate matter. The health impacts for airborne NH_3_-compounds are very uncertain and unit costs could be much smaller. Using typical emission factors [12] for NO_x_-N from fertilizer (0.2-0.6%) and NH_3_-N (1-3% for Calcium Ammonium Nitrate which is the most common chemical fertilizer in the EU) mean damage costs were derived for the EU per kg applied fertilizer N (Table 4). This result illustrates that although the potential health damage cost per unit of N emission in pollutant for NO_3 _(0.7 euro/kg of N) is much lower than for NO_x _and NH_3_, health damage values expressed per kg of added fertilizer-N are comparable because of the relatively high emission factor for nitrate. Consideration of the health cost of nitrate leaching from fertilizer and manure in agriculture, is therefore a relevant N-related externality that needs to be considered when defining the socially optimal input level of nitrogen in agriculture [40], in addition to externalities related to impacts of nitrogen use on ecosystems and greenhouse gas emissions. This will be further explored in a forthcoming paper.

## Conclusions

Health loss due to nitrate in drinking water is an issue under debate both in the scientific and policy arena. Estimates of associated health loss and potential welfare effects can help to evaluate current nitrate policies and measures. We derived a first and tentative estimate of a 3% increase of incidence of colon cancer for 11 EU member states due to nitrate in drinking water exceeding 25 mg/L, being half the legal US and EU limit of 50 mg/L. This health impact corresponds to an economic loss of 2.9 euro/capita/yr and of 0.7 euro per kg of NO_3_-N leaching. The cost of water treatment to abate exceedance of 25 mg/L ranges between 0.5 and 3 euro/capita/yr, indicating that these measures are beneficial for society. Average costs to prevent nitrate exceedance by reduced fertilizer use range between 0.6 and 2.7 euro/kg of N-fertilizer, and tend to be lower in regions with intensive fertilizer use. These values indicate that in these regions, reduction of fertilizer use will also likely create net benefits for society, particularly when drinking water production is vulnerable to nitrate leaching.

However, the epidemiological evidence for increased risk of colon cancer is weak or absent. Clearer negative or positive answers about associations between nitrate in drinking water and disease are reasonably to be expected from prospective case-control studies. In view of the magnitude of the potential health gain (2.9 euro/capita/year) improved epidemiological studies would certainly be worthwhile. Then, integrated cost-benefit assessment of nitrogen management, including all relevant impacts and measures, including those debated for nitrate in drinking water, may help to further improve current EU nitrogen policies from a precautionary approach.

## Abbreviations

CAFE: EU programme Clean Air for Europe; CBA: Cost Benefit Assessment; DALY: Disability Adjusted Life Year); ExternE: EU programme Externalities of Energy generation; EU: European Union (EU25; EU with 25 member states); GDP: Gross Domestic Product; NDMA: nitrosodimethylamine; QALY: Quality Adjusted Life Year; VYLD: economic Value of Life Year with Disability (Value of a YLD); US EPA: USA Environmental Protection Agency; VOLY: economic Value Of a Life Year; YLD: number of Years Lived with Disability per cancer case; YLL: number of Years of Life Lost.

## Competing interests

The authors declare that they have no competing interests.

## Authors' contributions

HJMG is an agro-environmental scientist and did the actual assessment. AR is one of the lead scientists in ExternE and contributed to the social cost approach. TMK is a toxicologist and provided input on medical and physiological aspects of colon cancer incidence. All authors read and approved the final manuscript.
